# Supplementary material for: A humanized IFN-γ mouse model reveals skin eschar formation, enhanced susceptibility and scrub typhus pathogenesis
Source: PLoS Pathog. 2026 Feb 11;22(2):e1013419. doi: 10.1371/journal.ppat.1013419 (PMC12912700; doi:10.1371/journal.ppat.1013419)
Supplement: S1 Table — All primer sequences are obtained from PrimerBank (https://pga.mgh.harvard.edu/primerbank/) or our previous publications. (DOCX) [file ppat.1013419.s001.docx]

| **S1 Table** |
| --- |

**Real-time PCR primers of murine genes**

Forward (5’ to 3’) Reverse (5’ to 3’)

*Ifngr1* CTGGCAGGATGATTCTGCTGG GCATACGACAGGGTTCAAGTTAT

*Ifngr2*  TCCTCGCCAGACTCGTTTTC GTCTTGGGTCATTGCTGGAAG

*Cxcl10* CCAAGTGCTGCCGTCATTTTC GGCTCGCAGGGATGATTTCAA

*Nos2* GTTCTCAGCCCAACAATACAAGA GTGGACGGGTCGATGTCAC

*Ifit1* CTGAGATGTCACTTCACATGGAA GTGCATCCCCAATGGGTTCT

*Ifit2* AGTACAACGAGTAAGGAGTCACT AGGCCAGTATGTTGCACATGG

*Gbp2* CTGCACTATGTGACGGAGCTA GAGTCCACACAAAGGTTGGAAA

*Gbp4* GGAGAAGCTAACGAAGGAACAA TTCCACAAGGGAATCACCATTTT

*Gbp5* CAGACCTATTTGAACGCCAAAGA TGCCTTGATTCTATCAGCCTCT

*Isg15* GGTGTCCGTGACTAACTCCAT TGGAAAGGGTAAGACCGTCCT

*Isg20* TGGGCCTCAAAGGGTGAGT CGGGTCGGATGTACTTGTCATA

*Stat1* TCACAGTGGTTCGAGCTTCAG CGAGACATCATAGGCAGCGTG

*Rsad2* TGCTGGCTGAGAATAGCATTAGG GCTGAGTGCTGTTCCCATCT

*Oas2*  TTGAAGAGGAATACATGCGGAAG GGGTCTGCATTACTGGCACTT

*Oas3* TCTGGGGTCGCTAAACATCAC GATGACGAGTTCGACATCGGT

*Oas1b* GGGCCTCTAAAGGGGTCAAG TCAAACTTCACTCCACAACGTC

*Oasl2* TTGTGCGGAGGATCAGGTACT TGATGGTGTCGCAGTCTTTGA

*Il6*  TAGTCCTTCCTACCCCAATTTCC TTGGTCCTTAGCCACTCCTTC

*Il27* CTGTTGCTGCTACCCTTGCTT CACTCCTGGCAATCGAGATTC

*Tnf* CCCTCACACTCAGATCATCTTCT GCTACGACGTGGGCTACAG

*Sele* ATGCCTCGCGCTTTCTCTC GTAGTCCCGCTGACAGTATGC

*Selp*  CATCTGGTTCAGTGCTTTGATCT ACCCGTGAGTTATTCCATGAGT

*Ifitm3* CCCCCAAACTACGAAAGAATCA ACCATCTTCCGATCCCTAGAC

*Icam1* GTGATGCTCAGGTATCCATCCA CACAGTTCTCAAAGCACAGCG

*Vcam1* AGTTGGGGATTCGGTTGTTCT CCCCTCATTCCTTACCACCC

*Endothelin-1* GCACCGGAGCTGAGAATGG GTGGCAGAAGTAGACACACTC

*Vegf* CTGCCGTCCGATTGAGACC CCCCTCCTTGTACCACTGTC

*Gapdh*  TGGAAAGCTGTGGCGTGAT TGCTTCACCACCTTCTTGAT

**Real-time PCR primers of human genes**

Forward (5’ to 3’) Reverse (5’ to 3’)

*IFNGR1* TCTTTGGGTCAGAGTTAAAGCCA TTCCATCTCGGCATACAGCAA

*IFNGR2* CTCCTCAGCACCCGAAGATTC GCCGTGAACCATTTACTGTCG
