# Supplementary figures and images for: A humanized IFN-γ mouse model reveals skin eschar formation, enhanced susceptibility and scrub typhus pathogenesis
Source: PLoS Pathog. 2026 Feb 11;22(2):e1013419. doi: 10.1371/journal.ppat.1013419 (PMC12912700; doi:10.1371/journal.ppat.1013419)

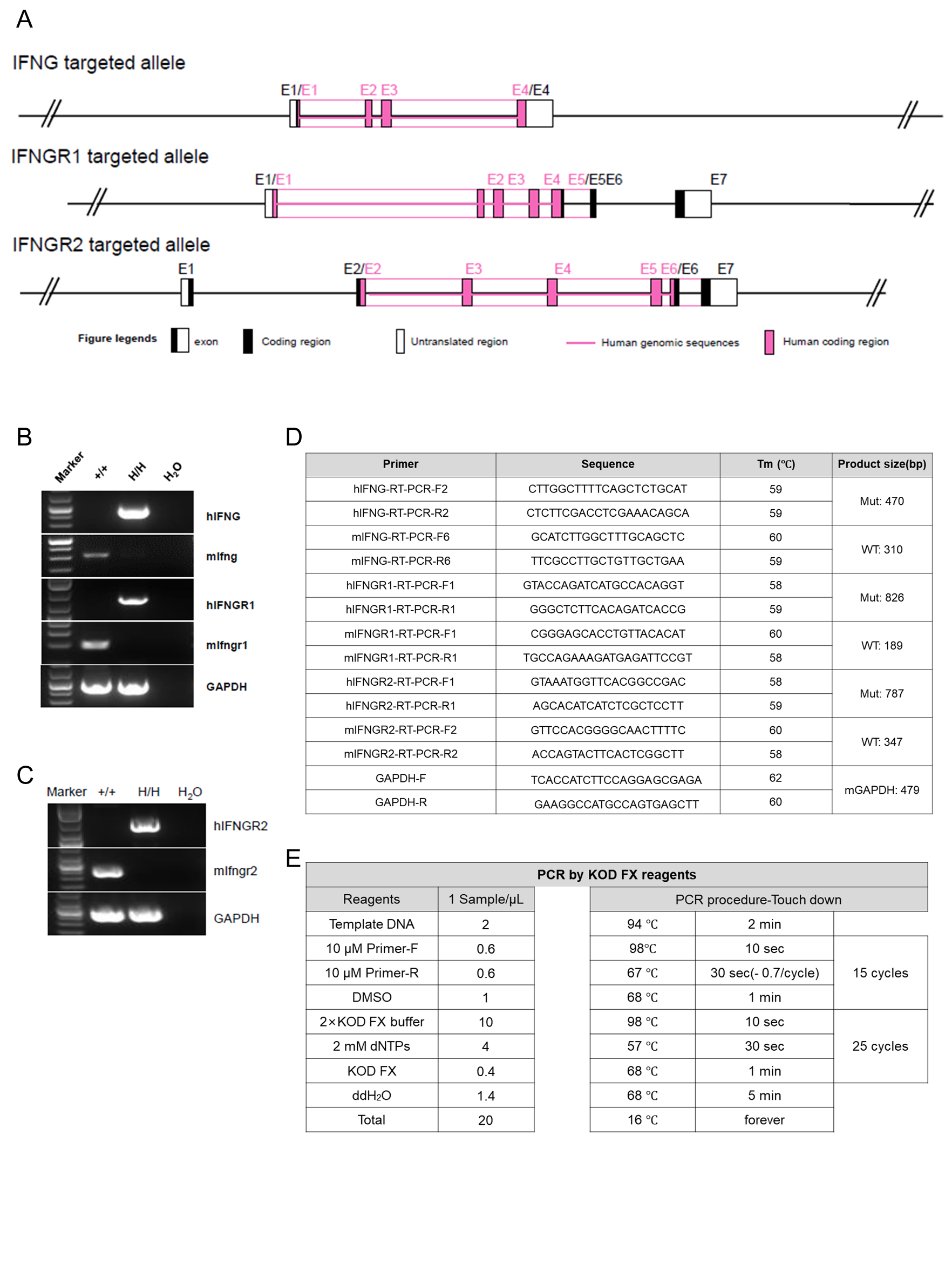

Supplement: S1 Fig — (A) Schematic strategy for generation of humanized IFNG, IFNGR1, and IFNGR2 alleles. (B) Ifng and Ifngr1 expression was analyzed in B-hIFNGR1/hIFNG mice by RT-PCR. Human Ifng and Ifngr1 mRNA were detectable in splenocytes of the homozygous B-hIFNGR1/hIFNG mice (H/H), but not in WT mice (+/+). (C) Human Ifngr2 mRNA was exclusively detectable in the small intestine of homozygous B-hIFNGR2mice (H/H), but not in that of WT mice (+/+). (D-E) PCR primers and experimental conditions were listed. (TIF) [file ppat.1013419.s002.TIF]

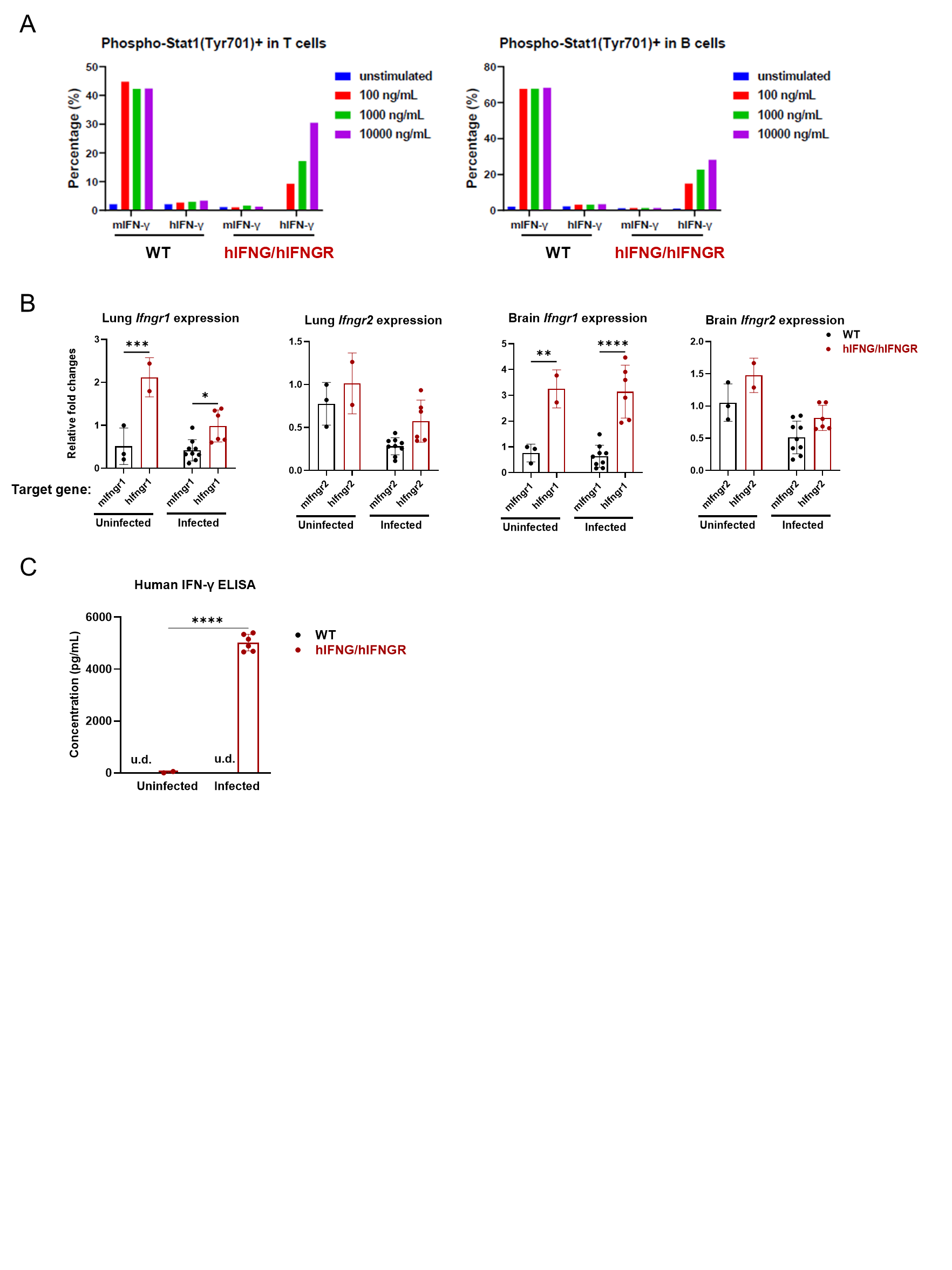

Supplement: S2 Fig — (A) Splenocytes were isolated from naïve hIFNG/hIFNGR mice, followed by mouse or human IFN-γ cytokine stimulation ex vivo. The phosphorylated STAT1 protein expression in T and B cells was analyzed by PhosFlow. (B) Mice were infected as in Fig 1. Brain and lung tissues were harvested for analyzing mouse and human Ifngr1 and Ifngr2 transcripts by qRT-PCR. Unpaired t-test was used for data analysis between two groups under either uninfected or infected condition. *, p < 0.05; **, p < 0.01; ***, p < 0.001; ****, p < 0.0001. (C) Serum samples were collected from WT and hIFNG/hIFNGR mice at 14 dpi. Human IFN-γ levels were measured by ELISA. One-way ANOVA with a Šídák’s multiple comparisons was used for data analysis. ****, p < 0.0001, u.d., undetectable. Data are presented as mean ± SD from three independent pooled experiments. Comparisons with no significant differences are not labeled. (TIF) [file ppat.1013419.s003.tif]

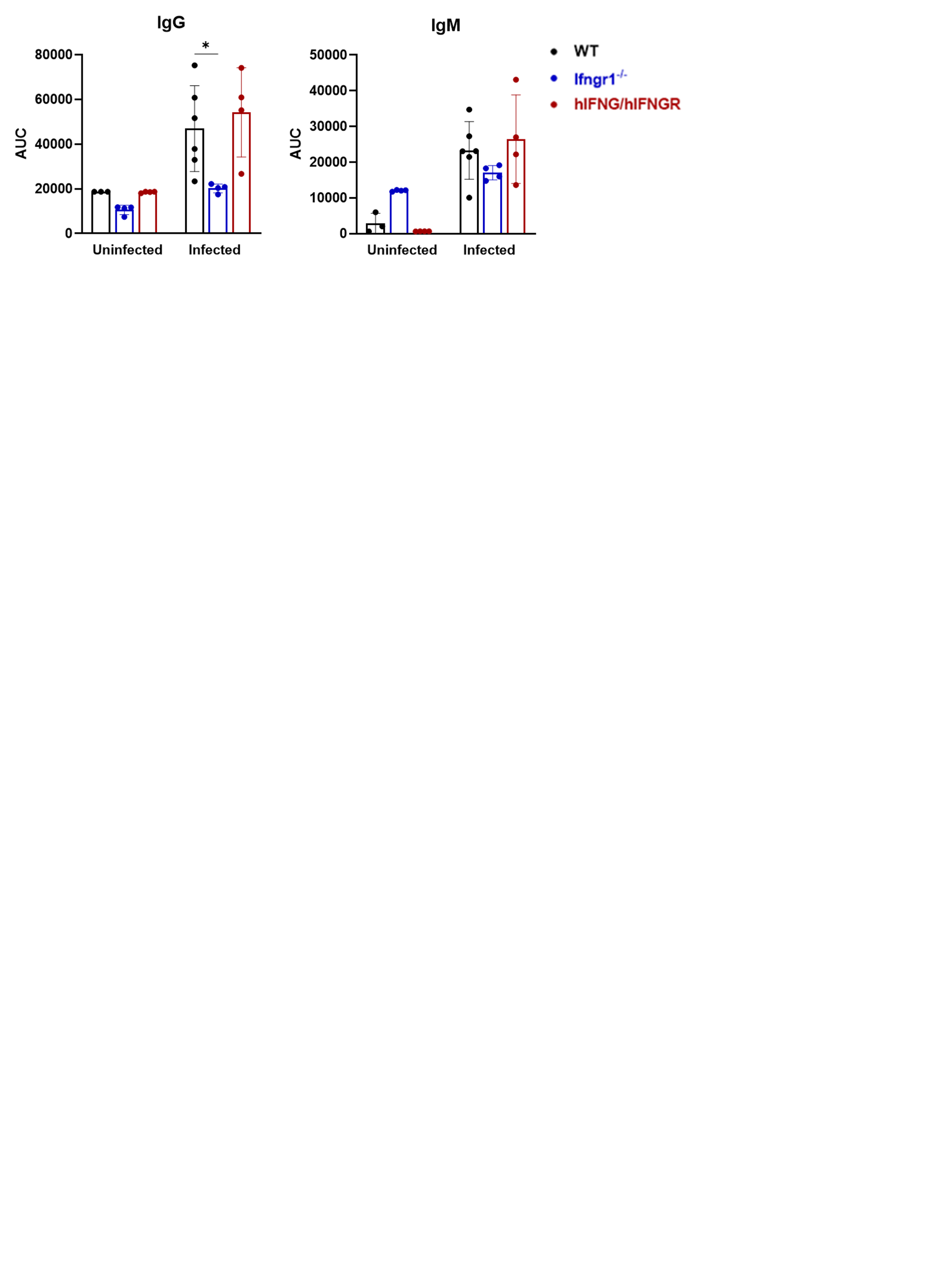

Supplement: S3 Fig — To measure relative amounts of IgM and IgG antibodies, mouse sera were collected at 14 dpi as shown in Fig 1 and then diluted to create a dilution curve. Uninfected mouse sera were used as controls. The area under the curve (AUC) is calculated from three pooled independent experiments and shown as mean ± SD. One-way ANOVA with a Šídák’s multiple comparisons was used for data analysis of infected groups. *, p < 0.05. (TIF) [file ppat.1013419.s004.TIF]

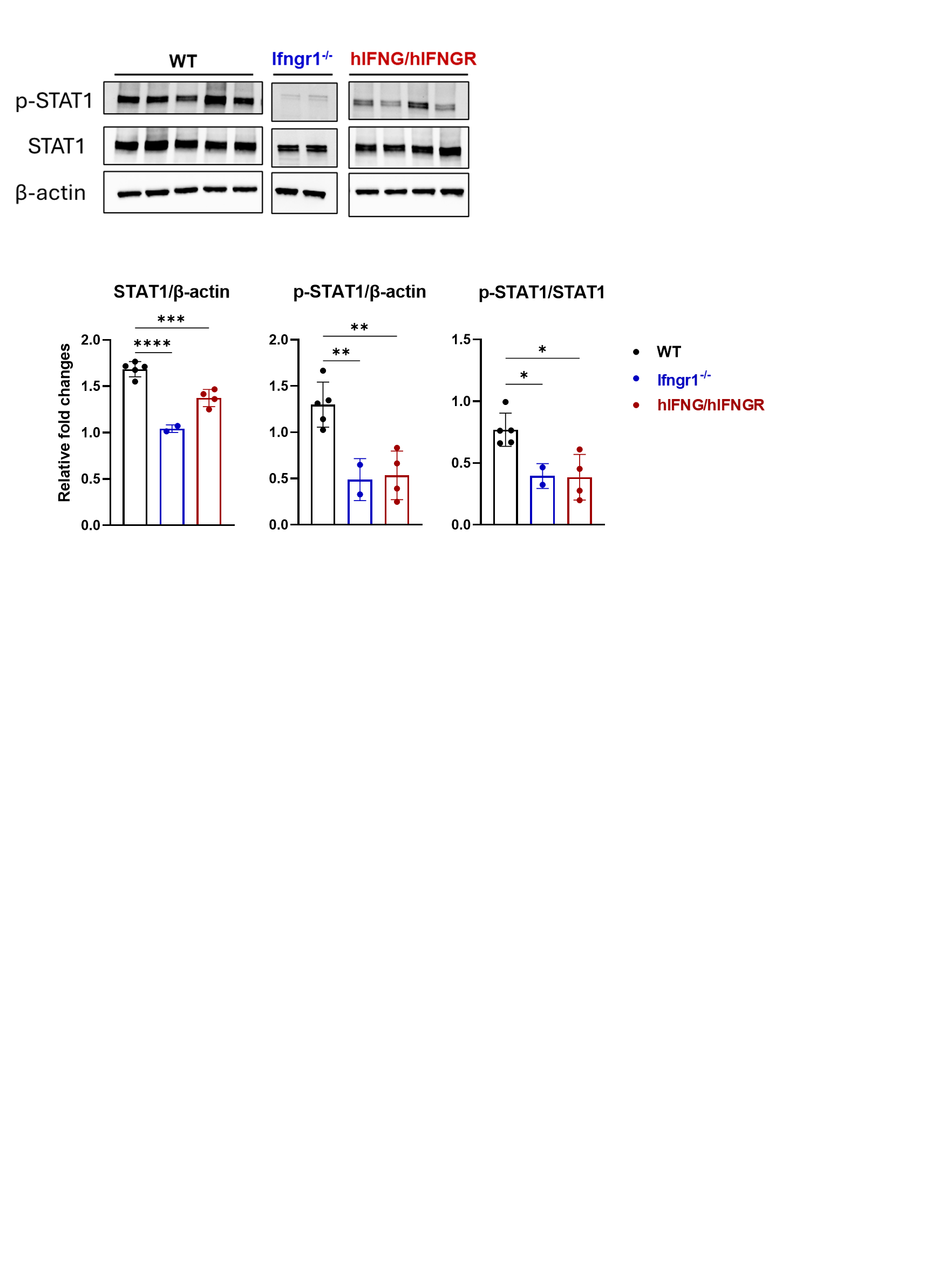

Supplement: S4 Fig — Mice were infected as described in Fig 1 and were euthanized at 14 dpi. Lung tissues were collected and analyzed for phospho-STAT1, total STAT1, and β-actin by western blot. Protein bands were visualized using an Amersham Imager 680, and signal intensities were quantified using ImageJ software. Data is shown as mean ± SD and analyzed by one-way ANOVA with a Šídák’s multiple comparisons test. *, p < 0.05; **, p < 0.01; ***, p < 0.001, ****, p < 0.0001. Comparisons with no significant differences are not labeled. (TIF) [file ppat.1013419.s005.TIF]

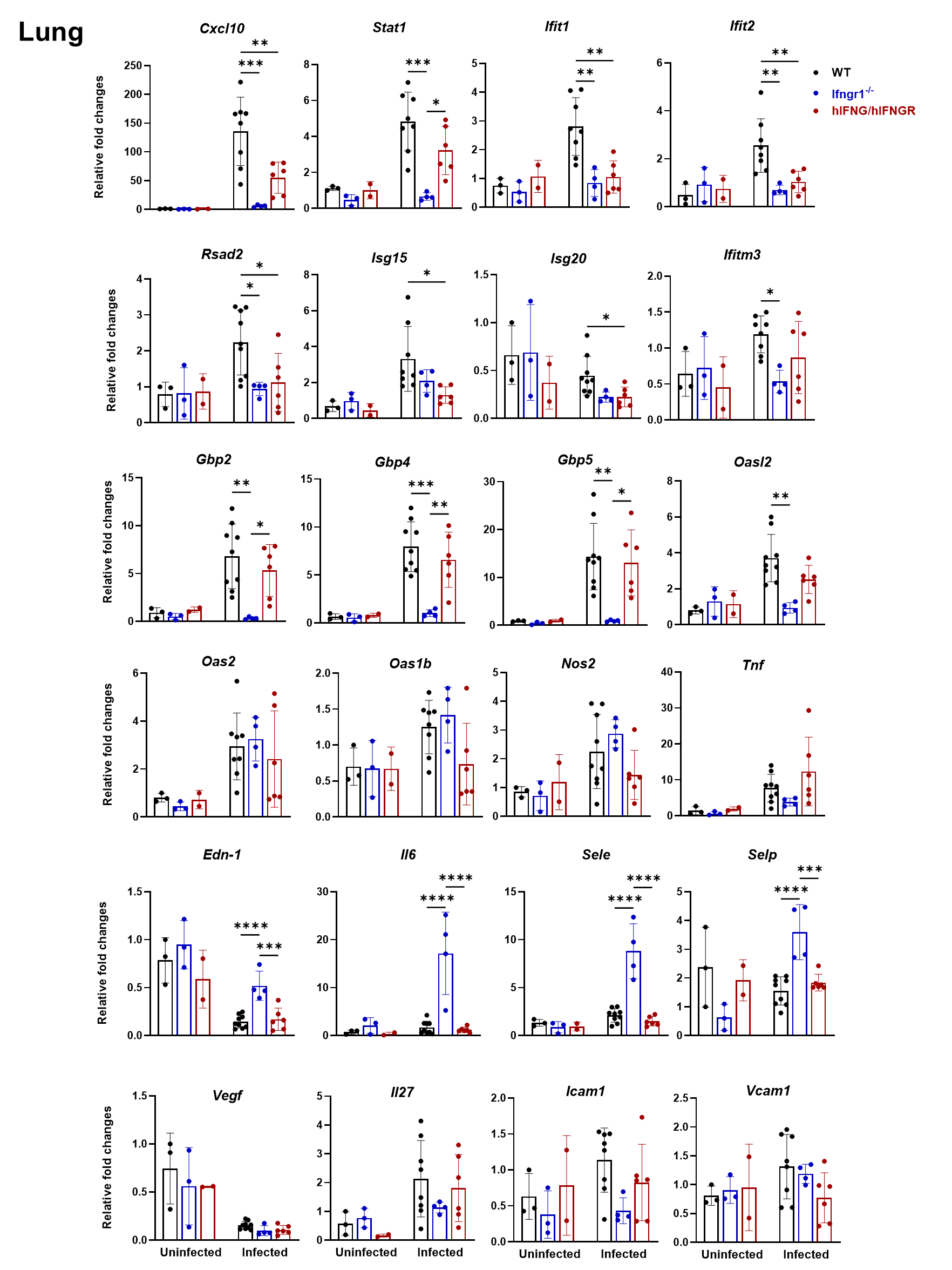

Supplement: S5 Fig — Mice were infected as described in Fig 1 and were euthanized at 14 dpi. The transcript levels of ISGs and inflammatory genes in the lungs were analyzed by qRT-PCR. Data is shown as mean ± SD from three pooled independent experiments. One-way ANOVA with a Šídák’s multiple comparisons test was performed for the infected groups. *, p < 0.05; **, p < 0.01; ***, p < 0.001. Comparisons with no significant differences are not labeled. (TIF) [file ppat.1013419.s006.TIF]

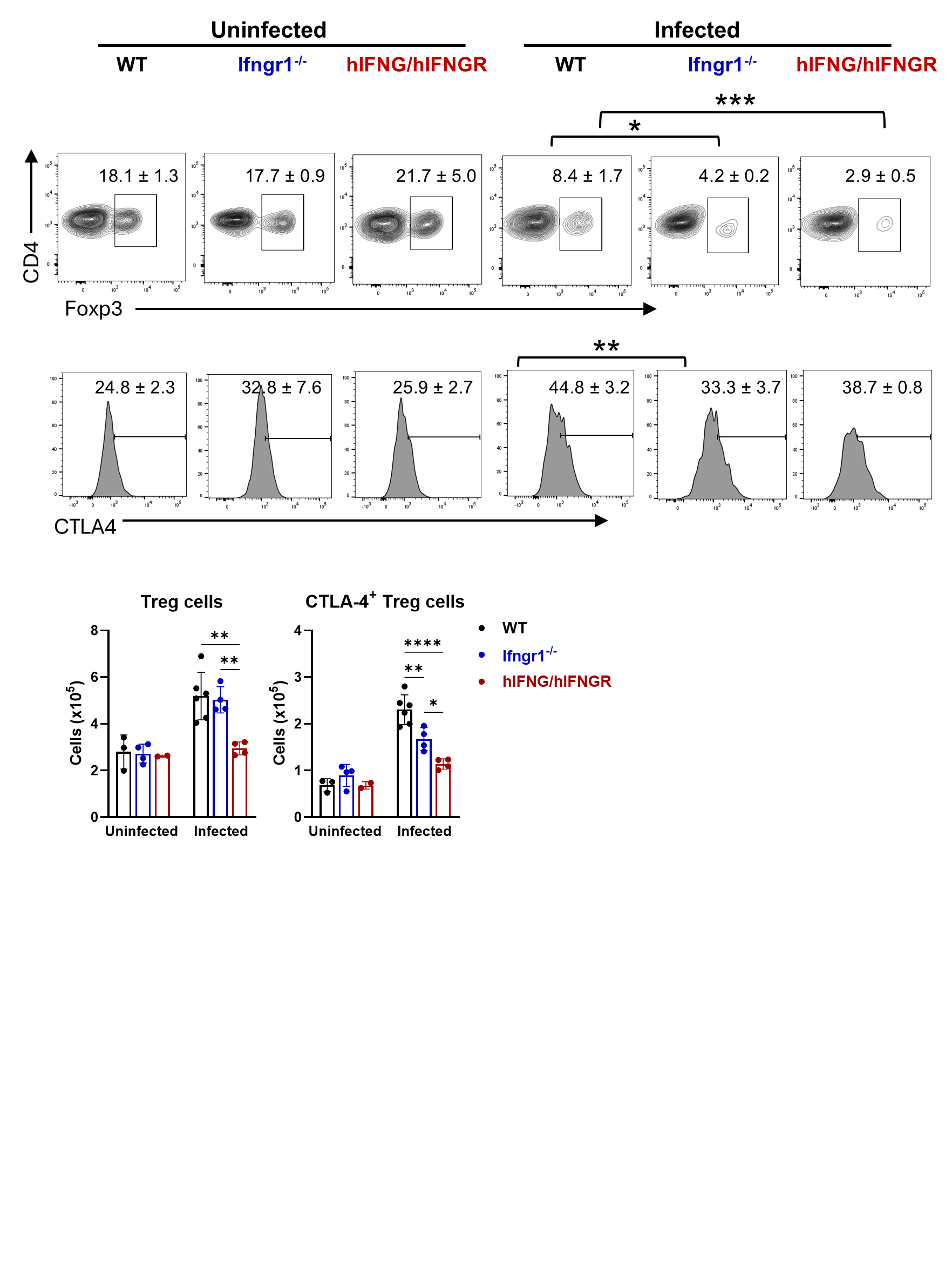

Supplement: S6 Fig — Mice were infected as described in Fig 1 and were euthanized at 14 dpi. Splenocytes were isolated for flow cytometric analysis of regulatory T (Treg) cells, which were identified by CD4+ Foxp3+. The expression of CTLA4 was further gated on CD4+Foxp3+ T cells. The percentages of cell populations were shown as mean ± SD on the flow cytometric images and the statistical analysis between infected groups were labeled. The absolute numbers of cell populations were calculated and were shown below the flow cytometric images. One-way ANOVA with a Šídák’s multiple comparisons was used for data analysis of infected groups. *, p < 0.05; **, p < 0.01; ***, p < 0.001; ****, p < 0.0001. Comparisons with no significant differences are not labeled. (TIF) [file ppat.1013419.s007.TIF]
