# Supplementary material for: A humanized IFN-γ mouse model reveals skin eschar formation, enhanced susceptibility and scrub typhus pathogenesis
Source: PLoS Pathog. 2026 Feb 11;22(2):e1013419. doi: 10.1371/journal.ppat.1013419 (PMC12912700; doi:10.1371/journal.ppat.1013419)
Supplement: Attachment — Submitted filename: Point-by-point reply 12_24_25 Ryan Comments.docx [file ppat.1013419.s008.docx]

Major concerns:

**Reviewer #1:**

***1****. The first major problem of this study is lack of validation of the “humanized mouse” that is used….There are no cited studies or data regarding how the mouse was constructed or whether the human IFN-g and IFN-g receptor are functional and expressed to the same degree as the native mouse genes. What parts of the human genes are swapped? Is the 5’ and 3’ UTR included? The promoter?*

Reply: We appreciate the reviewer for identifying this important weakness. In response, we have worked with Biocytogen to add a new supplementary figure detailing the generation of the mouse strains, along with the associated methodology. (Lines 150-191, S1 Fig, S2 Fig)

**2**. *There is no description about whether the protein products are functional and modified similarly.*

Reply: We thank the reviewer for this important question. In the original submission, we quantified human IFN-γ levels in uninfected and infected mice (S3C Fig). As expected, uninfected hIFNG/hIFNGR mice expressed only marginal levels of human IFN-γ, whereas infected hIFNG/hIFNGR mice produced robust amounts of human IFN-γ; human IFN-γ was undetectable in wild-type mice (Figure 3A). To address functional validation, we have added a new supplementary figure demonstrating that immune cells from hIFNG/hIFNGR mice respond to human IFN-γ stimulation, as evidenced by increased STAT1 phosphorylation (S3A Fig). To further confirm the functionality of IFN-γ downstream JAK–STAT signaling, we performed additional experiments analyzing STAT1 phosphorylation in lung tissues (S5 Fig). Our result showed that hIFNG/hIFNGR mice can active STAT1 signaling, although the magnitude is lower than WT mice. This data suggests the functional, but attenuated IFN-γ signaling in hIFNG/hIFNGR mice following *Ot* infection. We have revised the results and discussion accordingly. (Lines 398-401, 407-408, 454-461)

**3**. *They should validate that they make similar abundancies of IFN-g and the g-receptor. This will also require significantly more validation, for example showing that the hIFN-gamma receptor binds to the hIFN-gamma made in the mouse and elicits similar downstream cytokine signaling.*

Reply: Per the reviewer’s suggestion, we added a new supplementary figure demonstrating that recombinant human IFN-γ induces STAT1 phosphorylation in T and B cells isolated from hIFNG/hIFNGR mice (S3A Fig). Although p-STAT1 levels in hIFNG/hIFNGR mouse cells were lower than those in WT cells, they exhibited dose-dependent activation following IFN-γ stimulation. This finding is consistent with our tissue qRT-PCR data showing that *Ot* infection significantly increased interferon-stimulated gene (ISG) expression in hIFNG/hIFNGR mice, although the magnitude was lower than that observed in WT mice (Figure 4C and S6 Fig). There results indicate that human IFN-γ can bind the human IFNGR in hIFNG/hIFNGR mice and induce similar downstream signaling of ISG. However, the reasons why human IFN-γ induces a lower level of downstream signaling remain unclear. It is reported that co-expression of human IFNgR1 and IFNgR2 in mouse macrophages is necessary and sufficient for most IFN-γ-induced responses, but it is not sufficient to confer a generalized antiviral state (PMID: 8955096). There are some possibilities for this, such as the difference of receptor extracellular binding affinity, intracellular association with JAK1/JAK2 and efficient STAT1 recruitment and phosphorylation. There might also be some differences of post-translational modifications between human and mouse IFNGR as the reviewer mentioned. In addition, we provide the qRT-PCR data of receptors before and after infection. Our result suggests that hIFNG/hIFNGR mice express comparable or even higher levels of receptors (S3B Fig). Our goal of this study is to establish a susceptible animal model of scrub typhus, as WT mice are highly resistant to intradermal infection, which mimics the natural route of chigger transmission. hIFNG/hIFNGR mice exhibit functional but attenuated IFN-γ signaling and are susceptible to *Ot* infection, developing skin lesions that recapitulate a key diagnostic feature of human scrub typhus but are absent in WT mice, supporting their utility as a promising animal model for future studies. We revised the manuscript accordingly. (Lines 351-353, 606-626)

***4****. Regarding post-translational modifications (PTMs) on the humanized version – has this been examined by mass spectrometry? A brief search of the mouse and human IFNGR suggests that they have different PTM sites.*

Reply: We appreciate the reviewer’s insightful question regarding post-translational modifications. Currently, we do not have experimental data addressing this aspect. Given that this mouse strain is newly developed and remains incompletely characterized, further studies will be necessary to delineate potential differences in signaling transduction and post-translational regulatory mechanisms. Accordingly, we have revised the Discussion to acknowledge this limitation. (Lines 606-626)

***5****. There is no discussion about the regulation of human versus mouse IFNGR / IFN-g – are the genes regulated at the mRNA level? The protein level? Epigenetically?*

Reply: Both human and mouse IFNG/IFNGR signaling are similarly regulated at the transcriptional and protein levels. Human and mouse IFN-γ share only ~40% amino acid sequence homology and do not functionally cross-react, reflecting species-specific requirements for IFN-γ signaling. The IFN-γ receptor complex itself also differs between species, and effective signal transduction requires species-matched IFNGR1 and IFNGR2 components, with species-specific interactions involving the extracellular domains of these receptors (PMID: 1532657). Despite these differences, both human and mouse IFN-γ activate the conserved JAK–STAT signaling pathway. Importantly, human IFNGR expressed in mouse macrophages can bind human IFN-γ with an affinity comparable to that observed in human cells (PMID: 8955096), and ligand binding elicits canonical IFN-γ-responsive signaling. However, functional outcomes are different. Human IFN-γ fails to fully protect mouse cells from the cytopathic effects of encephalomyocarditis virus and vesicular stomatitis virus, whereas mouse IFN-γ provides robust protection (PMID: 8955096). These observations suggest that species-specific differences in downstream signaling efficiency exist beyond receptor binding. As noted by reviewer 1, such differences may involve post-translational regulation of IFNG/IFNGR signaling components. Actually, IFN-γ-induced STAT1 activation and IRF-mediated transcriptional programs drive extensive epigenomic remodeling that reshapes gene expression in immune cells, such as macrophages (PMID: 29921905). However, the different epigenetic regulation between human and mouse IFNG/IFNGR requires further investigation.

***6****. Figure 3 and Section in lines 320-336 start to address the above point by measuring transcripts of human vs mouse IFNGR, however the data appear to show that human IFNG1 and IFNG2 are only expressed at a 1 “relative fold change” – what does this mean? Wouldn’t a 1-fold change mean no change? The more important comparison to make is to ask how does the mRNA abundance of mIFNGR compare to hIFNGR? If hIFNGR is expressed 2x or even 10x less than the mIFNGR, this could explain the observed phenotypes, assuming that the protein products were functional.*

Reply: We thank the reviewer for this question and apologize for the confusion caused by the original presentation of these data. In response, we have replotted the data and moved it to S3B Fig. In this revised figure, we analyze the qRT-PCR relative transcriptional levels of *Ifngr* before and after infection to determine whether hIFNG/hIFNGR mice express receptor transcripts at levels comparable to those of WT mice. Our data showed that *Ifngr1* expression was higher in hIFNG/hIFNGR mice than in WT mice, whereas IFNGR2 expression was comparable between the two groups. These results suggest that the attenuated IFN-γ signaling observed in hIFNG/hIFNGR mice is unlikely to be due to reduced or impaired receptor expression.

***7****. In 3C, the most important comparisons are not performed – which would be to compare human IFN-g abundance to mouse IFN-g abundance by Western to determine whether the proteins are expressed to similar degrees in infected and uninfected scenarios, as compared to a control.*

Reply: We agree with the reviewer that IFN-γ concentrations should be directly measured. In the original version, both mouse and human IFN-γ levels were measured using either Bio-Plex or ELISA. We have replotted these data in our revised manuscript. As shown in Figure 3A, *Ot* infection resulted in increased mouse IFN-γ levels in WT mice, whereas mouse IFN-γ was not detected in hIFNG/hIFNGR mice. Conversely, S3C Fig demonstrates a significant increase in human IFN-γ levels in hIFNG/hIFNGR mice following infection, while human IFN-γ was absent in WT mice. Notably, hIFNG/hIFNGR mice produced higher levels of human IFN-γ compared with the levels of mouse IFN-γ detected in infected WT mice.

***8****. The second major problem is that throughout the manuscript, the appropriate control of Ifngr-/- was not used except in Figure 1. If the “humanized” mouse appears phenotypically identical to the IFNGR-/- mouse, is this humanized model a valid mouse model to use or should researchers just use the IFNGR-/- mouse?*

Reply: We thank the reviewer for this critical and insightful question. In response, we performed additional experiments using *Ifngr1*⁻/⁻ mice and have incorporated these new data into multiple figures. Notably, the phenotypes observed in *Ifngr1*⁻/⁻ mice differ from those in hIFNG/hIFNGR mice. First, *Ifngr1*⁻/⁻ mice are extremely susceptible to *Ot* infection, with even low inoculum doses resulting in 100% mortality within three weeks as we reported recently (PMID: 38743761). This severe outcome does not reflect the clinical course observed in most human scrub typhus cases, in which patients typically survive and often recover with appropriate immune responses. hIFNG/hIFNGR mice can develop skin lesions and systemic disease manifestations that more closely recapitulate key features of human scrub typhus. Second, *Ifngr1*⁻/⁻ mice lack intact IFN-γ signaling, a condition that does not occur in humans. By comparison, hIFNG/hIFNGR mice retain functional, albeit attenuated, IFN-γ signaling. Because IFN-γ signaling is essential for immune cell activation, including dendritic cell maturation, macrophage polarization, and interferon-stimulated gene induction, preservation of this pathway is critical for mechanistic studies of disease pathogenesis. Moreover, our new data indicate that *Ifngr1*⁻/⁻ mice exhibited impaired IgG response, might be suboptimal for evaluating humoral immune responses and vaccine candidates (S4 Fig). Collectively, these findings support the conclusion that the hIFNG/hIFNGR mouse strain represents a physiologically relevant and promising model for studying scrub typhus pathogenesis.

***9****. For example Figs 4 and 5: The IFNGR-/- control is not included, so it’s unclear whether these phenotypes are due to a dysfunctional hIFNg or just a lack of signaling. Line 377 “the results suggest that human IFN-g signaling confers weaker antibacterial activity compared to its murine counterpart” – based on what specifically? The authors haven’t shown that the hIFN-g is expressed at the same levels as the mouse IFN-g, so it could just be that there is reduced abundance of hIFN-g overall, and it’s slightly upregulated during infection.*

Reply: We thank the reviewer for pointing out the absence of *Ifngr1*⁻/⁻ control data. To address this concern, we performed additional experiments and have now included *Ifngr1*⁻/⁻-related data across all relevant figures. We also quantified both mouse and human IFN-γ levels in serum samples (Figure 3A and S3 Fig). These analyses demonstrate that hIFNG/hIFNGR mice can produce high levels of human IFN-γ following *Ot* infection. We apologize for the inappropriate statement regarding the antibacterial activity of human IFN-γ in the original version. This statement has been revised in the Results section (Lines 451-452).

**Reviewer #2**: (No Response)

**Reviewer #3:**

***1****. It is possible that human IFNGR 1/2 does not effectively activate mouse JAK-STAT under any condition. This study will be strengthened with the inclusion of a control demonstrating that JAK-STAT signaling is not impaired altogether in the humanized mouse and that the diminished ISG expression reflects a phenotype specific to O.t. infection in this mouse.*

Reply: We thank the reviewer for this important question. In response, we have added new supplementary data demonstrating that recombinant human IFN-γ induces STAT1 phosphorylation in immune cells isolated from humanized mice, indicating that IFN-γ signaling is functional in this model (S3A Fig). Notably, we observed that p-STAT1 induction in cells from humanized mice is quantitatively weaker than that observed in WT mice. This suggests the attenuated IFN-γ downstream signaling in humanized mice. In addition, our data show that *Ot* infection induces robust production of human IFN-γ in humanized mice, accompanied by increased expression of interferon-stimulated genes (ISGs) in infected tissues (S3C Fig, Figure 4). These findings are consistent with our previous reports demonstrating that *Ot* infection elicits a strong type I immune response in mice and patients (PMID: 25254971, 36566774, 28742087). The comparatively lower magnitude of ISG induction in humanized mice may reflect attenuated activation of the JAK-STAT pathway by human IFN-γ in the murine cellular context. This interpretation is further supported by prior studies showing that, although human IFN-γ can signal through human IFNGR transgenes in mouse cells and induce similar immune responses as that of mouse IFN-γ, human IFN-γ does not confer full protection against viral infection (PMID: 8955096). Therefore, it is likely that the diminished ISG induction may not be specific to *Ot* infection, but related to attenuated JAK-STAT signaling induced by human IFN-γ. Lastly, we added new *Ifngr1*⁻/⁻ control group data across all relevant figures. Consistent with our previous report (PMID: 38743761), *Ifngr1*⁻/⁻ mice are highly susceptible to *Ot* infection accompanied by greatly reduced ISG induction. Together, these observations suggest that IFN-γ signaling in humanized mice is functional but quantitatively attenuated relative to mouse IFN-γ signaling.

***2****. Are posttranslational JAK-STAT activation markers also reduced in humanized mice during O.t. infection?*

Reply: We thank the reviewer for this question. We have added new data of p-STAT1 levels in the lungs following infection (S5 Fig). Our data indicates that hIFNG/hIFNGR mice elicited functional, but attenuated IFN-γ signaling as compared to WT mice. This could be the reason why hIFNG/hIFNGR mice are more susceptible to *Ot* infection than WT mice.

***3****. It is unclear why human IFN-γ would cause such significantly different immune responses when compared to mice. The paper would benefit from added discussion on this topic.*

Reply: The reasons why human IFN-γ induces a lower level of downstream signaling remain unclear. It is reported that co-expression of human IFNgR1 and IFNgR2 in mouse macrophages is necessary and sufficient for most IFN-γ-induced responses, but it is not sufficient to confer a generalized antiviral state (PMID: 8955096). There are several possibilities for this, such as the difference of receptor extracellular binding affinity, intracellular association with JAK1/JAK2 and efficient STAT1 recruitment and phosphorylation. There might also be some differences of post-translational modifications between human and mouse IFNGR as reviewer 1 mentioned. Our goal of this study is to establish a susceptible animal model of scrub typhus, as WT mice are highly resistant to intradermal infection, which mimics the natural route of chigger transmission. hIFNG/hIFNGR mice exhibit functional but attenuated IFN-γ signaling and are susceptible to *Ot* infection, developing skin lesions that recapitulate a key diagnostic feature of human scrub typhus but are absent in WT mice, supporting their utility as a promising animal model for future studies. We revised the manuscript accordingly. (Lines 454-461, 606-626)

**Minor concerns:**

**Reviewer #1:**

***1****. The authors state in the abstract and introduction that “1 million cases occur annually, with more than 1 billion people at risk”….These numbers appear to be frequently reported in the Orientia literature but are in fact not supported by any epidemiology study. The authors should either provide strong epidemiology studies, which appears to not exist, or should remove these statements.*

Reply: We appreciate the reviewer for pointing out our inappropriate statement. Scrub typhus is still a neglected tropic disease. As we know, only five countries/regions-Japan, mainland China, South Korea, Taiwan, and Thailand have designated scrub typhus as a notifiable disease in their national surveillance systems (PMID: 38964725). This makes it difficult to accurately collect case data in endemic regions. According to a recent meta-analysis publication, which analyzed 663 articles from 29 countries/regions, the overall pooled seroprevalences of scrub typhus were 10.73% and 22.58% in healthy people and in febrile patients, respectively. We have revised this in our manuscript (Lines 77-79).

***2****. Line 35- It's confusing to say that animal models are needed to mimic infection via mites, and then the manuscript is not about using mites for infection.*

Reply: We apologize for this unclear statement. We have revised this accordingly.

***3****. Lines 305-307: The authors suggest that this humanized mouse model is immunocompetent, suggesting that their previous findings with an IFNGR-/- mouse is not immunocompetent? Are IFNGR-/- mice considered immunodeficient as compared to these hIFNGR/hIFNg mice?*

Reply: We agree with the reviewer that our original use of the term was imprecise. Mice lacking *Ifngr1* exhibit selective impairment in IFN-γ-dependent immune pathways rather than a global immunodeficiency, as seen in SCID or Rag knockout models. In contrast, hIFNG/hIFNGR mice retain functional IFN-γ signaling. We have removed the inappropriate description in the manuscript.

***4****. Discussion: Since this would be the first description of using this mouse model for an infectious disease study, what other infectious diseases or pathogens would this be useful for studying? A paragraph the potential broad utility of this model would be useful in the discussion for folks in the broader field outside of Orientia.*

Reply: We thank the reviewer for this thoughtful suggestion. Because IFN-γ signaling plays a critical role in host protection against many infections, we believe that this mouse strain may serve as a useful model for evaluating vaccines and therapeutics targeting pathogens that rely heavily on IFN-γ-mediated immunity such as rickettsial infection (PMID: 3106216, 2498207, 11504408) as well as other bacteria, protozoa, fungi and viruses (PMID: 38635718). WT murine models are often resistant to infection by many human pathogens, and IFN-deficient mouse strains (e.g., A129 and AG129) are therefore widely used to model severe disease. However, complete loss of IFN-γ signaling can result in profound abnormalities in both innate and adaptive immune responses, which may not accurately reflect the integrated immune responses observed in humans. This limitation can complicate mechanistic studies, vaccine candidate evaluation, and therapeutic screening. In contrast, this humanized mouse strain retains functional, albeit quantitatively attenuated, IFN-γ signaling and partially recapitulate human immune responses, thereby providing a more physiologically relevant platform for studying pathogen susceptibility and host-pathogen interactions. We have revised the manuscript accordingly (Lines 634-636).

***5****. Throughout the manuscript, referring to these mice as “humanized mice” is confusing and vague. There are hundreds of examples of “humanized mice”. The authors should call them “hIFNGR/hIFN-g mice” in the text and figures.*

Reply: We agree and have revised it accordingly.

**Reviewer #2:**

***1****. Line 353: As this statement is written, it suggests that WT mice had a significant increase in AMY levels during infection. According to the figure, this is not true when compared to the uninfected control WT mouse.*

Reply: We have corrected this accordingly.

***2****. Figure 1 and 2: It could be due to the quality of the figures, but I am unable to see the scale bar for the skin eschars in Figure 1C and histology images in Figure 2.*

Reply: We have revised this by improving the scale bar labeling in the figures.

***3****. Line 401-406: I believe the figure panels are incorrectly cited in the text.*

Reply: Thank you for pointing out this error. We have reorganized figures and corrected this.

***4****. Could the authors comment as to what immunological shift could be occuring around day 11 to prevent humanized mice from succumbing to infection like Ifngr1-/- mice?*

Reply: This is an excellent question. Owing to funding limitations and the high cost of purchasing humanized mice, we were unable to comprehensively characterize the full temporal dynamics of infection in this model. Nevertheless, existing data and our prior work provide a mechanistic framework for interpreting the observed phenotypes. *Ot* mainly targets endothelial cells and causes vascular damage. The severity of scrub typhus is strongly influenced by bacterial burden, which is controlled by efficient and timely immune activation. Early control of *Ot* following intradermal infection is highly dependent on host innate immunity, particularly IFN-γ signaling within the lymphatic system (PMID: 38743761). In WT mice, robust IFN-γ signaling effectively restricts bacterial replication in the draining lymph nodes, thereby limiting systemic dissemination. Subsequently, adaptive immune responses, especially CD8⁺ T cells further contribute to bacterial clearance (PMID: 28723951, 27606708), resulting in mild disease manifestations. In contrast, *Ifngr1*⁻/⁻ mice lack functional IFN-γ signaling and are therefore highly susceptible to *Ot* infection, developing markedly elevated bacterial burdens across multiple organs (PMID: 38743761). Humanized mice occupy an intermediate phenotype: they retain functional but quantitatively attenuated IFN-γ signaling and reduced anti-bacterial ISG expression. This diminished signaling permits greater bacterial dissemination and endothelial damage than in highly resistant WT mice, contributing to transient weight loss. Importantly, bacterial burdens in humanized mice remain substantially lower than those observed in *Ifngr1*⁻/⁻ mice, and these animals begin to recover by approximately day 12 post-infection. Collectively, these findings indicate that preserved IFN-γ signaling in humanized mice is sufficient to support partial bacterial control and recovery, highlighting the critical role of IFN-γ-mediated immunity in regulating disease severity.

***5****. As stated in the introduction, there is extensive antigenic diversity among Ot strains. Could the authors elaborate on information as to whether they believe this humanized mouse model could serve as a model across all Ot isolates?*

Reply: We recently reported that protective immunity against *Ot* infection is highly dependent on IFN-γ signaling. Using two *Ot* strains with differing virulence, Karp (highly virulent) and Gilliam (less virulent), we demonstrated that *Ifngr1*⁻/⁻ mice are susceptible to infection with both strains, indicating that IFN-γ-mediated protection is conserved across *Ot* strains (PMID: 38743761). Although humanized mice are less susceptible than *Ifngr1*⁻/⁻ mice, they develop pronounced disease manifestations, including characteristic skin eschar lesions, a key diagnostic feature of human scrub typhus that are not observed in WT mice. These findings suggest that humanized mice, which retain intact immune signaling, represent a physiologically relevant model for studying *Ot* infection. Further studies are warranted to systematically evaluate strain-specific differences in pathogenesis and immune responses using this model.

**Reviewer #3**:

***1****. Histology images are dark and have poor contrast. White and color balancing these images will ease interpretation by the reader.*

Reply: We have improved the color of histology images.

***2****. Fig 4A: Graph should be titled IL-1β; Fig 5A: Kidney is not capitalized; Fig 5B: Figure title capitalization should be consistent across panels*

Reply: We have addressed these issues and really appreciate all the comments from our reviewers. Given that IL-1β levels were consistently low and did not yield meaningful interpretation, this analyte has been removed from the revised figure.
