# Supplementary material for: A humanized IFN-γ mouse model reveals skin eschar formation, enhanced susceptibility and scrub typhus pathogenesis
Source: PLoS Pathog. 2026 Feb 11;22(2):e1013419. doi: 10.1371/journal.ppat.1013419 (PMC12912700; doi:10.1371/journal.ppat.1013419)
Supplement: Attachment — Submitted filename: Point-by-point 01312026.docx [file ppat.1013419.s009.docx]

Reviewer #1:

We appreciate reviewer 1 for the very helpful suggestions, and We have revised the manuscript to the best of our ability to address these issues.

*1. The only two drawbacks of the study is that replacing the mouse IFNGR/IFN-g with the human homologs results in significantly less STAT1 phosphorylation and dramatically less ISG activation, for many ISGs the signaling is more similar to the IFNGR knockout mouse. Thus, the question remains unclear as to whether this mouse has “functional IFN-g signaling” as mentioned in the abstract line 41. The abstract should be accurately reflect the findings – either that this mouse model has aberrant upregulation of ISGs, or something similar, as line 41 would suggest that it is an ideal model.*

**Reply**: We agree with the reviewer that hIFNG/hIFNGR mice display significantly reduced ISG expression compared with WT mice during infection. Our in vitro and in vivo data demonstrate that, in this hIFNG/hIFNGR strain, human IFN-γ, but not mouse IFN-γ can induce canonical downstream signaling. Therefore, we either delete the word “functional” or revised it as “functionally attenuated IFNg signaling” in our manuscript to make this more accurate.

*2. The second minor drawback is that certain statistics should be added, described below. Fig 1B should show the statistical comparisions between Ifngr-/- and hIFNGR/hIFNg at day 11 and the comparison of WT to hIFNGr/hIFN at day 14, even if they are not significant, as these are important to the entire argument about the relevancy of the mouse model. Similarly, 1D should show the statistical comparison of Ifngr-/- to hIFNGR/hIFNg even if not significant.*

**Reply**: We apologize for the lack of clarity in the original figure legends. In fact, comparisons among the indicated groups were performed, and no significant differences were detected. To maintain figure clarity, we did not label these nonsignificant comparisons as “ns.” We have now revised the figure legend to clearly explain the statistical analyses performed and how the results are displayed.

*3. It is confusing why Figure 2 the background is yellow and not white? The images look unclear to me. Similar comment – it is hard to discern from these images what the authors are trying to convey, perhaps include arrows pointing out the relevant areas of interest.*

**Reply**: We thank the reviewer for raising this question. The yellow background is likely due to imaging settings (e.g auto white balance) on the PC, as the slides appear normal when examined under the microscope. We have tried our best to revised the figures accordingly and added arrows as the reviewer suggested to highlight the areas of interest, particularly regions of inflammatory infiltration.

*4. Figure 3 and elsewhere – the* *statistics appear somewhat randomly chosen. Perhaps indicate NS if the comparisons are not significant, or upload a supplemental file with all of the comparisons made to each other and to the controls.*

**Reply**: We thank the reviewer for raising this question and apologize for the lack of clarity in the description of the statistical analyses. The statistical tests used were not chosen randomly across figures. For most datasets, we applied one-way ANOVA followed by a multiple-comparisons test. Because multiple experimental groups were included and some comparisons are not biologically meaningful (e.g., uninfected WT compared with infected Ifngr1^⁻/⁻^ mice), we primarily present comparisons among different mouse strains under the same condition (either uninfected or infected). In Figure 3, we additionally performed within-strain comparisons between uninfected and infected conditions. For other figures, uninfected groups exhibited much lower ISG expression or cell numbers; therefore, comparisons between uninfected and infected groups were not shown to avoid excessive labeling and to maintain figure clarity. We have revised the figure legends accordingly.

*5. Line 178: should say “were” instead of “was”.*

**Reply**: We thank the reviewer for this. This mistake has been corrected.

*6. Also line 183 should state what mouse genotypes they are referring to and what control genotypes were included.*

**Reply**: We thank the reviewer for this and have revised it.

*7. Lines 78-80 could mention that compelling epidemiological studies are lacking in the field.*

**Reply**: We have added this information in the manuscript.

*8. Line 109 should replace “symptoms” with “disease manifestations”*

**Reply**: We have added this information in the manuscript.
